# Endothelial Progenitors Exist within the Kidney and Lung Mesenchyme

**DOI:** 10.1371/journal.pone.0065993

**Published:** 2013-06-18

**Authors:** Sunder Sims-Lucas, Caitlin Schaefer, Daniel Bushnell, Jacqueline Ho, Alison Logar, Edward Prochownik, George Gittes, Carlton M. Bates

**Affiliations:** 1 Rangos Research Center, Children’s Hospital of Pittsburgh, University of Pittsburgh Medical Center, Pittsburgh, Pennsylvania, United States of America; 2 Division of Nephrology, Department of Pediatrics, Children’s Hospital of Pittsburgh, University of Pittsburgh School of Medicine, Pittsburgh, Pennsylvania, United States of America; 3 Section of Hematology/Oncology, Children’s Hospital of Pittsburgh, University of Pittsburgh School of Medicine, Pittsburgh, Pennsylvania, United States of America; 4 Department of Microbiology and Molecular Genetics, University of Pittsburgh Medical Center, Pittsburgh, Pennsylvania, United States of America; 5 University of Pittsburgh Comprehensive Cancer Center, Pittsburgh, Pennsylvania, United States of America; 6 Division of Pediatric General and Thoracic Surgery, Children’s Hospital of Pittsburgh, University of Pittsburgh Medical Center, Pittsburgh, Pennsylvania, United States of America; National Cancer Institute, United States of America

## Abstract

The renal endothelium has been debated as arising from resident hemangioblast precursors that transdifferentiate from the nephrogenic mesenchyme (vasculogenesis) and/or from invading vessels (angiogenesis). While the Foxd1-positive renal cortical stroma has been shown to differentiate into cells that support the vasculature in the kidney (including vascular smooth muscle and pericytes) it has not been considered as a source of endothelial cell progenitors. In addition, it is unclear if Foxd1-positive mesenchymal cells in other organs such as the lung have the potential to form endothelium. This study examines the potential for Foxd1-positive cells of the kidney and lung to give rise to endothelial progenitors. We utilized immunofluorescence (IF) and fluorescence-activated cell sorting (FACS) to co-label Foxd1-expressing cells (including permanently lineage-tagged cells) with endothelial markers in embryonic and postnatal mice. We also cultured FACsorted Foxd1-positive cells, performed *in vitro* endothelial cell tubulogenesis assays and examined for endocytosis of acetylated low-density lipoprotein (Ac-LDL), a functional assay for endothelial cells. Immunofluorescence and FACS revealed that a subset of Foxd1-positive cells from kidney and lung co-expressed endothelial cell markers throughout embryogenesis. *In vitro*, cultured embryonic Foxd1-positive cells were able to differentiate into tubular networks that expressed endothelial cell markers and were able to endocytose Ac-LDL. IF and FACS in both the kidney and lung revealed that lineage-tagged Foxd1-positive cells gave rise to a significant portion of the endothelium in postnatal mice. In the kidney, the stromal-derived cells gave rise to a portion of the peritubular capillary endothelium, but not of the glomerular or large vessel endothelium. These findings reveal the heterogeneity of endothelial cell lineages; moreover, Foxd1-positive mesenchymal cells of the developing kidney and lung are a source of endothelial progenitors that are likely critical to patterning the vasculature.

## Introduction

The renal endothelium has been largely debated as arising from trans-differentiating resident hemangioblast precursors (vasculogenesis) [1] and/or from invading vessels (angiogenesis) [2], [3], [4]. The existence of a resident precursor for renal endothelial cells is further supported by the recent finding that Osr1-positive cells in the early intermediate mesoderm, precursors to all renal lineages, also gave rise to Flk-1 positive cells within the kidney; however, a renal lineage giving rise to endothelial cells has not been identified. The renal stroma, characterized by expression of Foxd1, has been shown to give rise to many different cell types including mesangial cells of the glomerulus as well as many other vascular supportive cells including fibroblasts, pericytes, vascular smooth muscle cells and renin cell precursors [5], [6]; however, it has not been reported to give rise to endothelial cells.

In the lung, endothelial cell precursors are thought to arise from the pulmonary mesenchyme via the process of vasculogenesis [7]. As is true in the kidney, there is a Foxd1 positive subpopulation in the developing pulmonary mesenchyme that has not been well characterized. Preliminary investigation of the Foxd1-positive cells in the lungs revealed that they similarly expressed pericyte markers [8]. There are no reports of endothelial cell progenitors arising from Foxd1-positive mesenchymal cells in the lung.

We hypothesize that the Foxd1 positive cells in the kidney and lung give rise to endothelium. Using *Foxd1EGFPcre* transgenic mice, we detected co-expression of green fluorescent protein (GFP: stromal marker) and endothelial markers in subsets of kidney cells at different embryonic stages by fluorescent activated cell sorting (FACS) and immunofluorescence (IF). Functionally, embryonic Foxd1/GFP-positive sorted renal stromal cells differentiated into tubular networks that expressed endothelial markers in an *in vitro* endothelial tubulogenesis assay and were able to endocytose acetylated low-density lipoprotein (Ac-LDL), which is a function specific to endothelial cells. Ultimately, the Foxd1-positive renal cortical stroma gives rise to a portion of the endothelium that populates the peritubular capillaries. In the developing lung, we also observed that a subset of Foxd1-positive mesenchymal cells co-expressed endothelial cell markers and that Foxd1 positive cells had the ability to behave as endothelial cells *in vitro*. Many surviving lineage tagged Foxd1-positive pulmonary cells expressed endothelial cell-specific markers in post-natal lungs. These results indicate that Foxd1-positive mesenchyme gives rise to a subset of the endothelium in the kidney and lung.

## Methods

### Animals

We used the transgenic *Foxd1EGFPcre* mouse line that expresses GFP and cre recombinase in the renal stroma [9] and a population of cells in the lung mesenchyme [8]. In order to permanently label and track the fate of the Foxd1-expressing cells, we bred *Foxd1EGFPcre* mice with GT Rosa CAG reporter mice (tdTomato) that express red fluorescent protein (RFP) in all cre positive derivatives [10]. The University of Pittsburgh Institutional Animal Care and Use Committee approved all experiments.

### Genotyping

Briefly, tail clippings and/or embryonic tissues were collected and genomic DNA was isolated. Polymerase chain reaction (PCR) amplification was used to identify all genotypes. The primers used to detect the *Foxd1EGFPcre* allele were: forward 5′-TCTGGTCCAAGAATCCGAAG-3′ and reverse 5′-GGGAGGATTGGGAAGACAAT-3,′ which showed a band at 450 base pairs (bp), while cre-negative mice had no band. The primers utilized to detect tdTomato were wildtype forward 5′-AAGGGAGCTGCAGTGGAGTA-3′, wildtype reverse 5′-CCGAAAATCTGTGGGAAGTC-3′, which showed a band at 297 bp, and mutant forward 5′-CTGTTCCTGTACGGCATGG-3′ and mutant reverse 5′-GGCATTAAAGCAGCGTATCC-3, ′ which showed a single band at 196 bp.

### Tissue Collection and Immunohistochemistry

For frozen sections, whole embryos, kidneys and lungs were fixed in 4% paraformaldehyde (PFA) and then dehydrated in sucrose and embedded in OCT medium. Sections were cut at 8 µm on a cryostat and stored at −20°C. For section IF, embryonic or isolated tissue sections were blocked in a 10% bovine serum albumin/donkey serum solution in PBS and incubated with primary antibodies including PECAM (catalog #553370, BD Biosciences, San Jose, CA), Erg (catalog #EPR3864, Epitomics, Burlingame, CA), Flk1 (catalog #550549, BD Biosciences), CD144/VE-cadherin (catalog #550548, BD Biosciences), Meca-32 (pan-endothelial, catalog #550563, BD Biosciences), Thrombomodulin (BDCA-3, catalog #AF3894, R&D Systems, Minneapolis, MN) and von Willibrand factor (vWF, catalog #AB7356, Millipore, Temecula, CA) overnight at 4°C. Sections were incubated with various secondary antibodies for one hour, washed, mounted and visualized with an upright Leica fluorescent microscope (Leica Microsystems, Buffalo Grove, IL). For whole mount immunofluorescence, organs were removed and placed into 4% PFA in PBS overnight, dehydrated through to 100% methanol, and stored at −20°C. Embryonic kidneys and lungs were rehydrated through a graded methanol series to 0.1% Tween in PBS (PBST). After blocking in 10% donkey serum in PBST for 1 hour at room temperature, tissues were incubated with 1∶100 concentrations of the following antibodies: anti-calbindin (catalog #C9848, Sigma-Aldrich, St Louis, MO), anti-PECAM (catalog #553370, BD Biosciences) anti-Foxd1 (catalog #sc47585, Santa Cruz Biotechnology, Santa Cruz, CA) and/or anti-Six2 (catalog #11562-1-AP, Proteintech, Chicago, IL) primary antibodies at 4°C overnight. The tissues were then washed extensively in PBST and subsequently incubated with 1∶100 concentrations of the following secondary antibodies: donkey anti-goat Alexa Fluor-488 (catalog #A11055, Invitrogen, Carlsbad, CA), goat anti-rabbit Alexa Fluor-594 (catalog #A11080, Invitrogen) or donkey anti-rat Alexa Fluor 647 (catalog #712-605-150, Jackson Immunoresearch, West Grove, PA). The kidneys and lungs were then extensively washed, mounted, and visualized with an Olympus confocal microscope (Center Valley, PA).

### Fluorescently Activated Cell Sorting (FACS)

For the FACS experiments only cre positive embryos were utilized. Subsequently, between 3-6 embryos were pooled from any one litter. For each time point three separate experiments were performed. Embryonic kidneys and lungs were removed at various developmental time points (E13.5-18.5) and were then placed into collagenase (0.03% collagenase in PBS) for 10 minutes at 37°C while shaking. The organs were then titurated through a 25-gauge syringe to make a single-cell suspension and run through a 40 µm cell strainer (catalog # 22-363-547, Fisher Scientific, Pittsburgh, PA). The cells were immunostained with endothelial (PECAM (catalog #551262 or 561410 or 561813, BD Biosciences), Flk1 (catalog #560070 or 561259, BD Biosciences) and CD-144/VE-cadherin (catalog #562242, BD Biosciences)) and pericyte (alpha-smooth muscle actin (catalog #C6198, Sigma)(αSMA) and CD73 (catalog #561543, BD Biosciences), CD13 (catalog #558744, BD Biosciences) and CD44 (catalog #560569, BD Biosciences)) conjugated antibodies at a concentration of 1∶20 and then sorted by the appropriate wavelengths. For the analysis of the early time points a minimum of 100,000 cells were analyzed, while for the older embryonic and adult tissues up to 2,000,000 cells were analyzed.

### Tubulogenesis Assay

These assays were performed as described [11]. Briefly, flow sorted cells were gated conservatively and collected as those expressing GFP only (Foxd1-positive), endothelial cell markers only (PECAM or Flk1) or both GFP and endothelial markers. The cells were placed in 24 well plates that had been coated with Cultrex (matrigel, catalog #3432-005-01, R&D Biosystems, Mill Valley, CA) and contained endothelial specific medium (catalog #CC-3156, Lonza, Walkersville, MD) with at least 10,000 cells per well. As a positive control human umbilical vein endothelial cells (HUVECs) were utilized as these cells are known to readily form endothelial-like tubules. The cells were grown for one week, fixed in 4% PFA, and immunostained for PECAM that was visualized with an inverted fluorescent microscope (Jenco, Portland, OR).

### Ac-LDL Assay

A specific function of endothelial cells is the ability to endocytose Ac-LDL; thus, we performed this assay on isolated Foxd1-positive cells from the embryonic kidney and lung as previously described [12]. Briefly, FACsorted GFP positive cells were placed onto coverslips that had been coated with gelatin, and were subsequently grown in a hypoxic environment in endothelial cell-specific medium for 5 days, which drives differentiation toward an endothelial cell phenotype [11]. The cells were then incubated with FITC-tagged Ac-LDL (catalog #L23380, Invitrogen) for one hour. Cells were fixed in 4% PFA and the nucleus was stained with DAPI and mounted on slides. Presence or absence of FITC label in the cytoplasm of the cells was observed with an upright microscope (Leica Microsystems, Buffalo Grove, IL).

## Results

### The Developing Renal Stroma Contains Cells that Co-express Endothelial Markers

**Figure 1:**
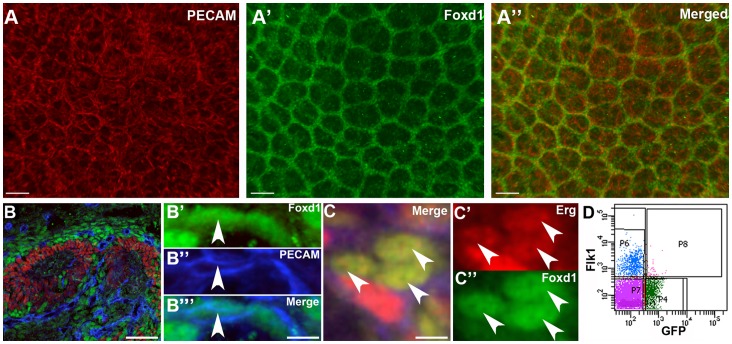
Immunofluorescence and FACS analysis reveals cells that co-express stromal and endothelial markers. **A–A’’**. Wholemount immunofluorescence reveals that PECAM positive vessels (A, red) are present within the Foxd1 positive renal stroma (A’, green) on the merged image (A’’). **B.** IF in the cortex of an E18.5 kidney shows that developing endothelial cells stained with PECAM (blue) are present in the stroma stained with Foxd1 (green) but not in nephron precursors stained with Six2 (red). **B’–B’’’.** Higher power views from “B” reveal expression of the stromal marker Foxd1 in the nucleus (green, arrowhead) and PECAM in the cytoplasm (blue) of the same cell. **C–C’’.** High power image of IF at E18.5 shows co-expression of the stromal marker Foxd1 (green) and the endothelial marker Erg (red) in nuclei of some renal cells (arrowhead). **D.** Flow sorted kidneys from E18.5 *Foxd1EGFPcre* mouse shows co-expression of GFP (Foxd1-positive stromal marker, X axis) and Flk1 (Y axis) in a subset of cells (P8). Scale bars A = 50 µm, B and C = 10 µm.

To investigate the relationship between the renal stroma and the developing endothelia in the embryonic kidney, we first performed wholemount immunofluorescence in E18.5 kidneys for Foxd1 and PECAM (endothelial marker). As shown, an extensive network of PECAM positive cells were embedded within the Foxd1-positive renal stroma (Figure 1A). Section immunofluorescence for PECAM, Foxd1 and Six2 (nephron progenitor marker) at E18.5 revealed that all PECAM positive cells were present only in stroma and excluded from nephron progenitors (Figure 1B); furthermore a subset of cells with Foxd1-positive nuclei appeared to co-express PECAM in the cytoplasm (Figure 1B). To confirm that some Foxd1-positive cells co-expressed EC markers, we performed immunolabeling with Foxd1 and Erg, a nuclear endothelial cell marker. As shown, Foxd1 and Erg were co-expressed in a subset of cells, strongly suggesting that a portion of the renal endothelium arises from renal cortical stromal cells (Figure 1C).

To complement the immunostaining studies showing apparent co-expression of Foxd1 and endothelial cell markers, we performed FACS analysis using *Foxd1EGFPcre* mice, which expresses GFP in the renal stroma under the direction of the Foxd1 promoter. First, we performed FACS analysis on cell suspensions from E13.5, E15.5, and E18.5 kidneys and examined expression of GFP and the endothelial marker Flk1. At each developmental stage, we observed three distinct populations of cells, GFP (Foxd1) positive stromal cells, Flk1 positive cells, and GFP/Flk1 double-positive cells (Figure 1D and Table S1). We then performed FACS analysis in E13.5-E18.5 kidney cell suspensions with other endothelial cell markers, PECAM and VE-cadherin. As with Flk1, we detected double- positive (GFP/endothelial marker) cells at each developmental time point (Table S2 and not shown). Since some Flk1-expressing cells outside of the kidney have been shown to differentiate into non-endothelial cells (i.e. pericytes and vascular smooth muscle) [13], we also performed FACS analysis in E13.5, E15.5, and E18.5 kidneys with Flk1 and with α smooth muscle actin (αSMA) (smooth muscle marker) or CD73 (pericyte marker); at no time point was there overlapping expression of Flk1 and αSMA or CD73 (Figure S1 and not shown). Taken together, the immunostaining and FACS analyses strongly suggest that there is a sub-population of Foxd1-positive renal stromal cells that give rise to endothelium in the kidney.

### Renal Stromal Cells have the Capacity to Differentiate into Endothelial-like Cells *in vitro*

**Figure 2:**
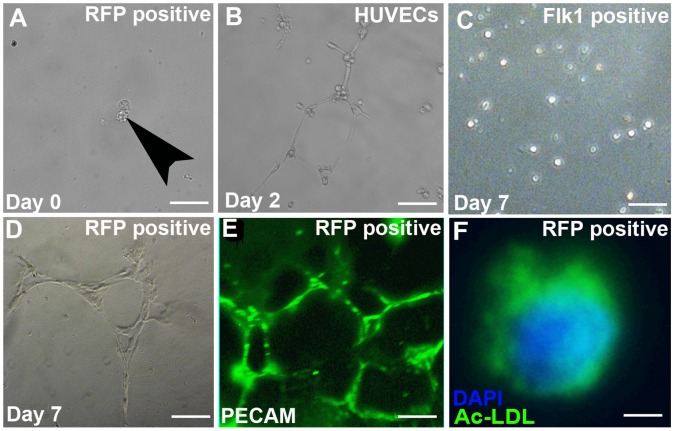
Renal stromal cells can differentiate into cells with endothelial like properties. **A–E.** Tubulogenesis assay**: A.** *Foxd1EGFPcre* positive sorted cells appear rounded when cultured at day zero (arrowhead). **B.** HUVECs (positive controls) develop the endothelial-like tubules after two days in culture. **C.** After 7 days of growth, the Flk1-positive populations remain rounded demonstrating that they had lost the ability to form endothelial-like tubules. **D–E**: After 7 days of growth, Foxd1-positive cells readily formed endothelial like tubes (D) and expressed PECAM by IF (E). **F.** Acetylated LDL assay**:** Under hypoxic conditions, cultured FACsorted Foxd1-positive renal stroma cells endocytosed Ac-LDL (green), which is a function specific to endothelial cells (blue = DAPI). Scale bars A–E = 50 µm, F = 5 µm.

We next tested the capacity of isolated renal stromal cells to behave like endothelial cells *in vitro*. First, we tested their capacity to form tube-like structures in semisolid medium, as happens with other endothelial cells [11], [14], [15]. We FACsorted E15.5 *Foxd1EGFPcre* kidney suspensions and cultured Flk1 positive cells, Foxd1/GFP-positive stromal cells or dual positive cells (as above). At day zero the three cell populations looked similar with round uniform cells (Figure 2A and not shown). After two days of culture, the positive control HUVEC cells formed endothelial-like tubular networks (Figure 2B). The Flk1 positive cells remained rounded up and did not form tubes throughout the 7-day culture period (Figure 2C). Conversely many of the Foxd1/GFP positive stromal cells elongated to form an endothelial-like tubular network (Figure 2D). In addition, while the Foxd1/GFP positive cells were initially PECAM-negative (not shown), they became PECAM-positive after they formed the tubules (Figure 2E). Cells that were double positive for endothelial and stromal markers formed tubular networks that were less robust than the Foxd1 population (not shown). To complement the tubulogenesis assay, we tested the ability of the GFP-positive renal stromal cells subjected to hypoxia to endocytose Ac-LDL, which is a function specific to endothelial cells [16]. We observed that the cultured stromal cells were able to endocytose Ac-LDL (Figure 2F). Thus the *in vitro* tubulogenesis and Ac-LDL assays revealed that Foxd1-positive stroma could be driven to behave like endothelial cells.

### Foxd1-positive Stromal Derivatives Differentiate into Peritubular Capillary Endothelial Cells

**Figure 3:**
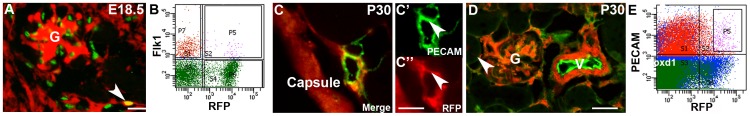
Stromal derivatives form endothelium that is present in peritubular capillaries. **A.** IF in E18.5 *Foxd1cre*CAG mouse section reveals RFP stromal derivatives (red) that co-label with Erg (green) in a peritubular capillary (arrowhead); in the glomerulus (G), the stromal-derived cells form the mesangium (red) but do not express Erg (green). **B.** Flow sorted kidneys from E18.5 *Foxd1cre*CAG reporter mice show co-expression of RFP (Foxd1-stromal derivative, X axis) and Flk1 (Y axis) in a subset of cells (P5). **C-C’’.** IF in a P30 *Foxd1cre*CAG kidney section reveals a peripheral peritubular capillary with co-expression of RFP stromal derivatives (red) and PECAM (green) (arrowhead). **D.** IF from a P30 *Foxd1cre*CAG kidney section shows a glomerulus (G) and major vessel (V), that express RFP stromal derivatives in the mesangium and vascular smooth muscle (red), respectively, but that do not co-express RFP and PECAM (green) in endothelium in those tissues. **E.** Flow sorted kidneys from P30 *Foxd1cre*CAG reporter mice show that many RFP stromal derived cells (X axis) co-express PECAM (Y axis) as shown in P5. Scale bars A = 30 µm, C = 20 µm, D = 50 µm.

**Figure 4:**
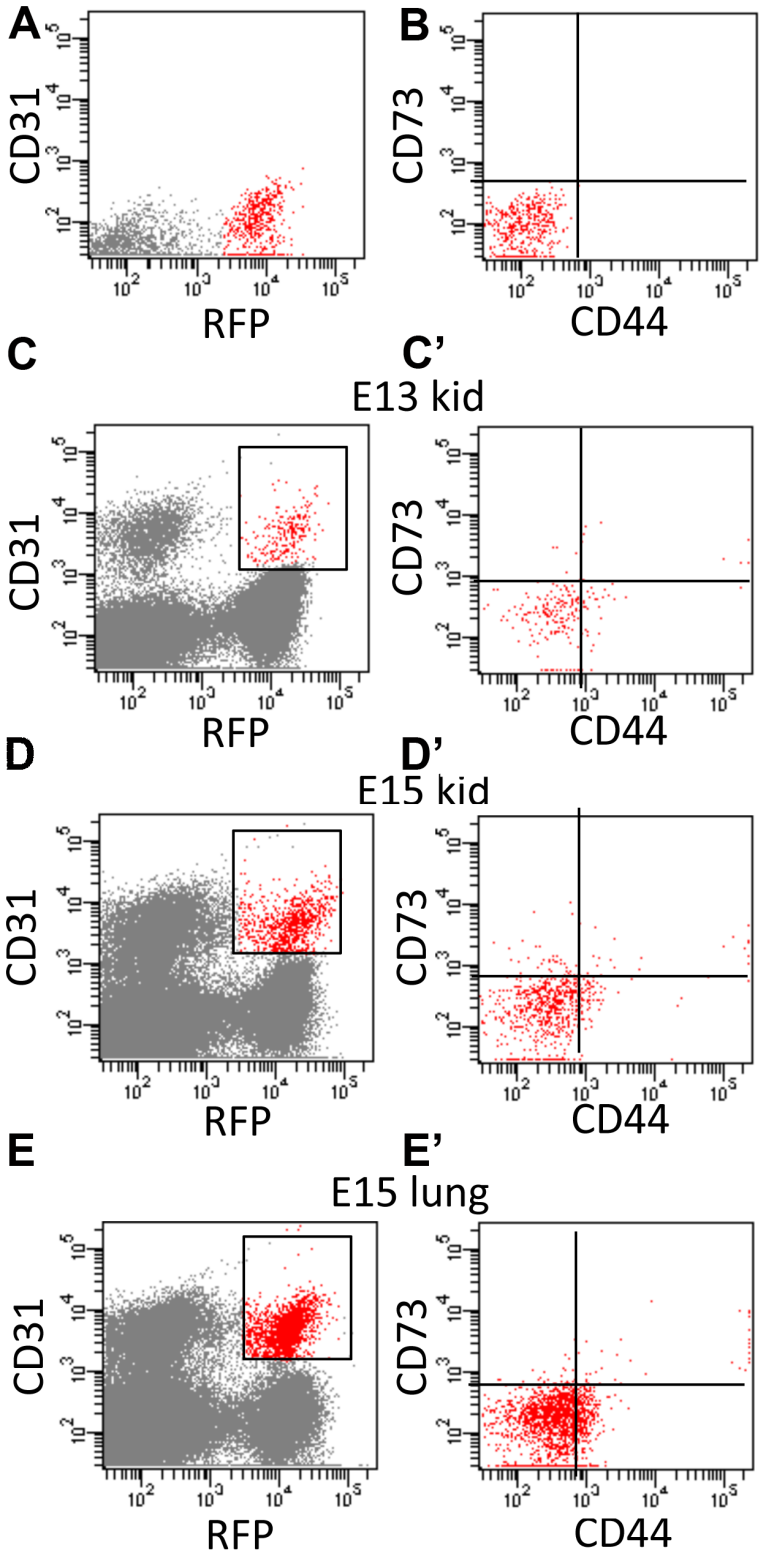
The majority of embryonic stromally derived endothelium does not express pericyte markers. Embryonic tissues FACS sorted using a back-gating strategy. This involved identifying the *Foxd1cre*CAG/Endothelial cells (box surrounding cells in B, C and D) and gating these against the pericyte specific markers CD73 and CD44 (B’, C’ and D’). **A.** Representative single color control for RFP-positive cells, the red dots represent the RFP positive cells. **B.** Representative FACS plot for control cells that are negative for CD73 and CD44, the red dots represent control cells that are negative for pericyte markers. **C.** E13.5 *Foxd1cre*CAG kidney cells stained with PECAM showing that 0.2% of the cells are Foxd1 derived endothelium (box). **C’.** Cells from “C” were then stained for pericyte markers (CD73 and CD44) and back gated against the pericyte markers, 75.3% of the cells that are endothelial-positive were pericyte negative. **D.** E15.5 kidney sample showing that 0.2% of the cells are Foxd1 derived endothelium (box). **D’.** Cells from “D” were then back gated against pericyte markers (CD73 and CD44), 84.0% of the cells that were endothelial positive were pericyte negative. **E.** E15.5 lung sample showing that 0.4% of the cells are Foxd1 derived endothelium (box). **E’.** Cells from “E” were then back gated against pericyte markers (CD73 and CD44), 83.4% of the cells that are endothelial positive are pericyte negative.

To determine the fate of the renal stromal cells that differentiate into endothelium, we bred the *Foxd1EGFPcre* with a GT Rosa CAG reporter mouse that permanently labeled the renal stroma and all of the descendants with RFP (*Foxd1cre*CAG*)* and first performed immunostaining and FACS analysis in embryonic kidneys for RFP and endothelial markers. By immunostaining, we detected RFP stromal derivatives that overlapped with Erg in E18.5 peritubular capillary endothelium (Figure 3A); although RFP stromal derivatives were also present in the glomerular mesangium, none of the RFP-positive cells in the glomerulus co-expressed Erg, suggesting that the none of the glomerular endothelium was of Foxd1-stromal origin. We also performed FACS analysis at various embryonic time points, after first gating out red blood cells and debris based on the size of the cells (Figure S2). To confirm that the sorted cells were single cell suspensions we then performed a doublet discriminator (allowing us to determine based on the size of the cells the forward and side scatter for an individual cell). As can be seen from the FACS plots (Figure S2) the vast majority of the cells were single cells. At various embryonic time points, we detected a population of RFP/Flk1 double positive cells by FACS analysis (Figure 3B and Table S1). To validate this finding we also performed the same FACS analysis using PECAM and found corroborating results (Table S2). Given that renal capillary endothelial cells lie adjacent to pericytes, we performed a rigorous back gating strategy to further confirm that the RFP and PECAM (CD31) double positive cells were really endothelial cells (and not pericytes). After removing debris and adhered cells with a doublet discriminator, we sorted out RFP/CD31 double positive cells in E13.5 and E15.5 kidneys (Figure 4). We then performed additional FACS analysis on the double positive cells to determine the percentage that expressed either CD44 or CD73, pericyte markers. As shown, the vast majority of the RFP/CD31 positive cells from embryonic kidneys did not express the CD44 pericyte marker.

We then examined *Foxd1cre*CAG P30 kidneys for co-expression of Foxd1 derived cells (permanently labeled with RFP) with endothelial cell markers. Consistent with the embryonic data, dual label immunostaining at P30 revealed co-labeling of the RFP and PECAM in peritubular capillaries (Figure 3C). Similarly, the P30 glomerular mesangium was RFP-positive, but none of glomerular ECs were RFP positive, suggesting that glomerular endothelium is not derived from the stroma (Figure 3D). In addition, the smooth muscle around major vessels within the P30 kidney was RFP-positive but the endothelium in these vessels was not (Figure 3D). We performed additional co-labeling studies with a battery of other endothelial markers, including Erg, VE-cadherin (CD144), Meca32, Thrombomodulin, and vWF. As shown, we identified RFP-positive stromal cell derivatives that co-expressed each of these endothelial cell markers (Figure S3). To quantitate the number of stromal cells that gave rise to endothelial cells and the number of endothelial cells of stromal origin, we performed FACS analysis of RFP and PECAM. We observed that 14.3% of the surviving RFP labeled Foxd1 stromal-derived cells became PECAM-positive endothelial cells and that 9.7% of the P30 renal endothelium arose from the Foxd1 stroma (Figure 3E and Table S3). Considering that all of the Foxd1 stromal-derived endothelial cells were excluded from the glomeruli and large vessels, a significant percentage of the P30 peritubular capillary endothelium was derived from the Foxd1 stroma.

### A Foxd1-positive Cell Population Exists in the Lung and Gives Rise to Endothelial Cells

**Figure 5:**
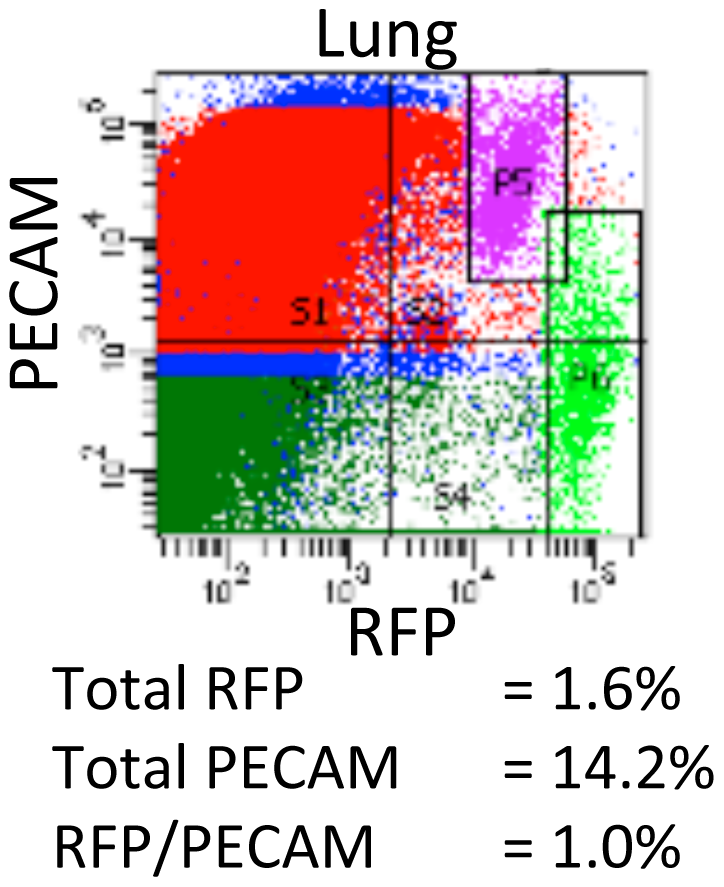
The adult lung contains Foxd1-derived endothelium. Representative FACS plot of adult lung showing a population of stromally derived endothelium (box, P5).

To determine whether Foxd1 positive cells in other organs could give rise to endothelial cells, we examined lung, heart and liver for RFP expression in *Foxd1cre*CAG mice. Heart and liver had negligible numbers of RFP expressing cells (and therefore Foxd1 derivatives) (not shown). However, lung had a significant population of RFP cells. Furthermore, FACS analysis revealed that the adult lung had a significant percentage of RFP-positive cells that also expressed the endothelial marker, PECAM (Figure 5). To confirm the presence of endogenous Foxd1 in the developing lung we performed real time PCR in comparison to the kidney. Here we found that the lung contained Foxd1-positive cells at E15.5 although Foxd1 was more abundant in the kidney (Figure S4).

### The Embryonic Lung also Contains Endothelial Progenitors Derived from the Foxd1 Stroma

**Figure 6:**
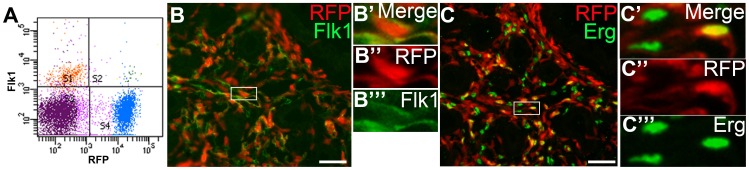
Foxd1-positive derivatives express markers of endothelium in the lung at E15.5. **A.** FACS sorting of a *Foxd1cre*CAG E15.5 lung shows co-expression of RFP (Foxd1-expressing cells- X axis) and Flk1 (Y axis) in a number of cells (panel S2). **B.** IF of an E15.5 *Foxd1cre*CAG lung reveals co-expression of RFP-positive Foxd1 derivatives (red) and the endothelial marker Flk1 (green) (box). **B’–B’’’.** High power images of panel B (box) confirms co-expression of RFP positive Foxd1 derivatives (red, nucleus) and Flk1 (green, cell membrane) in some pulmonary cells **C.** IF of *Foxd1cre*CAG E15.5 lung reveals co-expression of RFP-positive Foxd1 derivative (red) and nuclear endothelial marker Erg (green) (box). **C’–C’’’.** High power images of panel C (box) shows co-expression of the RFP-positive Foxd1 derivatives (red) and Erg (green) in nuclei of some pulmonary cells (yellow). Scale bar = 50 µm.

**Figure 7:**
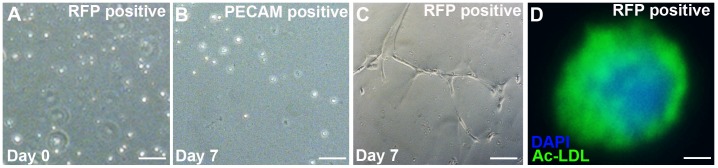
Foxd1 positive lung cells differentiate into endothelial-like tubules and take up Ac-LDL. **A–C.** Tubulogenesis assay: **A**. *Foxd1EGFPcre*/GFP positive sorted cells are rounded when cultured at day zero. **B.** After 7 days of growth, the PECAM positive populations remain rounded having lost the ability to form endothelial-like tubules. **C.** After 7 days of growth, *Foxd1EGFPcre*/GFP positive cells readily formed endothelial-like tubular networks. **D.** Acetylated LDL assay: Under hypoxic conditions, *Foxd1EGFPcre*/GFP cells have endocytosed Ac-LDL (green) (blue = DAPI). Scale bars A–C = 50 µm, D = 5 µm.

We then determined whether the embryonic lung had Foxd1-expressing cells with the potential to give rise to endothelium (like the kidney) or if the Foxd1-positive cells migrated in from an external source. Utilizing *Foxd1cre*CAG mice, we detected a significant RFP (Foxd1 derived) population as well as RFP/Flk-1 double positive cells by FACS analysis at E15.5 (Figure 6A). To localize these Foxd1 derived endothelial progenitors, we performed immunostaining for RFP and endothelial markers in E15.5 lungs. We detected co-expression of cytoplasmic Flk1 (Figure 6B) and nuclear Erg (Figure 6C) in a sub-population of cells surrounding the pulmonary epithelium. To confirm that the double positive cells truly represented endothelial cells and not pericytes, we performed a rigorous back gating strategy in E15.5 lungs similar to what we did in the kidney. As in the kidney, the vast majority of the RFP/CD31 positive cells did not express either CD73 and CD44 (Figure 4) or CD73 and CD13 (Figure S5). Finally, we tested the capacity of isolated Foxd1 cells from E15.5 lung to form tubular networks and endocytose AcLDL. Similar to the kidney, cultured Foxd1-expressing cells from the lung formed tubular structures and endocytosed Ac-LDL (Figure 7).

## Discussion

While the origin of the endothelium of the kidney has been a subject of debate, it has long been thought that an endogenous progenitor cell likely existed. Despite its plasticity, to our knowledge, the renal stroma has never been shown to give rise to endothelial cells. Using a combination of FACS analysis and immunostaining, we identified that a subpopulation of Foxd1-positive renal stromal cells expressed markers of endothelial cells. Furthermore, isolated Foxd1 renal stromal cells had the capacity to behave like endothelial cells *in vitro*, as seen in tubulogenesis and Ac-LDL uptake assays. Endocytosis of acetylated LDL is a function that is specific for endothelial cells and is a process that is mediated by a scavenger cell pathway of LDL metabolism that exists only within endothelial cells [12]. In addition, *in vitro* tubulogenesis assays and Ac-LDL uptake assays have been shown to predict the ability of tumor associated endothelial cells to differentiate into endothelial cells and integrate into tumor vasculature *in vivo* [11]. Permanent lineage tracing studies by FACS analysis and immunostaining with multiple markers and rigorous back gating confirms that a significant portion of the peritubular capillary endothelium of the kidney arose from the Foxd1 positive stroma. In examining other organs with Foxd1 cell populations, we determined that the lung (which develops in an analogous manner to the kidney) has a significant percentage of its endothelium that arises from Foxd1-positive cells.

The origin of the endothelium in the kidney is a subject of conjecture. Studies have shown that sprouting angiogenesis via the major renal vessels plays a significant role in formation of the kidney endothelium, giving rise to the major vessels and the glomerular capillaries [2], [5]. However, prior lineage-tracing experiments have determined that a significant proportion of the renal endothelium is derived from a resident precursor that was assumed to be from the metanephric (nephrogenic) mesenchyme [2]. While the Foxd1 renal stroma had been shown to give rise to many vascular supportive cells as well as the interstitium of the kidney [4], it was not thought to give rise to endothelium. Our findings clearly demonstrate the presence of stromally derived endothelium in the kidney. Interestingly, the *in vitro* studies suggest that the cells with the greatest potential to form vascular networks are those that are only Foxd1 positive, as opposed to those that were positive for both Foxd1 and endothelial markers (or those that were only Flk1 positive that could form no networks). Perhaps commitment toward an endothelial cell fate somehow diminishes the ability to form networks. In the case of the double positive cells, it may also be that the low plating density (due to low numbers of cells) diminished their ability to form networks. Alternatively, the committed cells may be more dependent on signals from the *in vivo* environment than the more plastic non-committed Foxd1-positive cells. Given that a large number of endothelial cells were not derived from the Foxd1 renal stroma, it is quite possible (if not likely) that there are other resident endothelial cell progenitors in the developing kidney such as hemangioblasts that have been suggested by others [4].

Given that we found that the Foxd1 population in the embryonic kidney could give rise to endothelium, we interrogated whether Foxd1 cells in other developing organs had the same potential. Interestingly, there appear to be few Foxd1 derivatives in the heart and the liver, whereas there are in the lung; furthermore, it appears that they form endothelial cells *in vitro*. The lung develops in a manner that is very analogous to the kidney (branching epithelium within mesenchyme). Perhaps the Foxd1-expressing cells in the lung represent a stromal mesenchymal compartment similar to that in the kidney. These data also confirm previous studies suggesting heterogeneity in the origin of the renal and pulmonary endothelium [2], [3], [6], [17], [18], [19], [20]. While the renal stroma gives rise to a portion of the peritubular capillary endothelium, they do not differentiate into glomerular or large vessel endothelium. These latter endothelial cells may originate via sprouting angiogenesis from invading vessels or as noted previously, from other unidentified resident precursor cells. Exactly how the Foxd1 derived endothelium interacts with the endothelial progenitors from other origins and angiogenic vessels to form the vast and complex vascular network of the kidney is unknown. Perhaps, the Foxd1 derived cells act as “instructive” cells, directing the invading angiogenic vessels so that they are able to establish proper connections with the vasculogenic networks. In any case the identification of endothelial precursor pools is critical to our understanding of the complex interactions that exist between angiogenesis and vasculogenesis that are paramount to normal organogenesis.

## Supporting Information

Figure S1 **Non-overlapping expression of Flk1 and pericyte or muscle markers in embryonic kidney. A.** Representative FACS plot from E15.5 kidney cells showing that there are no cells that co-express the endothelial marker Flk1 (Y axis) and the smooth muscle marker αSMA (Y axis) (panel R2). **B.** Representative FACS plot from E15.5 kidney cells showing that there are no cells that co-express the endothelial marker Flk1 (Y axis) and the pericyte marker CD73 (X axis) (panel Q2).
(TIF)

Figure S2 **Gating out of debris and doublet discriminator. A.** Representative FACS plot showing the gating strategy (box) used to eliminate the debris and red blood cells from E15.5 *Foxd1cre*CAG kidney single cell suspensions. SSC-A = side scatter-area, FSC-A = forward scatter-area. **B.** Representative FACS plot showing the gating strategy used for forward scatter (box) to eliminate adherent cells. FSC-W = forward scatter width, FSC-H = forward scatter – height. **C.** Representative FACS plot showing the gating strategy used for side scatter (box) to eliminate adherent cells clinging together. SSC-W = side scatter – width, SSC-H = side scatter- height. The red dots in all panels represent the distribution of the double-positive GFP (Foxd1)/Flk1 cells within the population of gated cells.
(TIF)

Figure S3 **Immunofluorescence showing expression of multiple endothelial markers in a subset of Foxd1 derivatives in E18.5 peritubular capillaries. A–A’’’.** RFP-positive cell derived from the Foxd1-expressing renal stroma (A) co-expresses the membrane endothelial marker CD144 (A’) and the nuclear endothelial marker Erg (A’’) as shown on the merged image (A’’’). **B–B’’’.** RFP-positive cell derived from the Foxd1-expressing renal stroma (B) co-expresses the membrane endothelial marker Meca32 (B’) and the nuclear endothelial marker Erg (B’’) as shown on the merged image (B’’’). **C–C’’’.** RFP-positive cell derived from the Foxd1-expressing renal stroma (C) co-expresses the membrane endothelial marker Thrombomodulin (C’) and the endothelial marker PECAM (C’’) as shown on the merged image (C’’’). **D–D’’’.** RFP-positive cell derived from the Foxd1-expressing renal stroma (D) co-expresses the Weibel-Palade bodies endothelial specific marker vWF (D’) and the endothelial marker PECAM (D’’) as shown on the merged image (D’’’).
(TIF)

Figure S4 **Representative real time PCR showing the presence of Foxd1 in the kidney and lung.** Gapdh was used to generate the delta CT. Kidney samples showed expression after 23 cycles while the lung took 29 cycles showing that relatively the kidney contains more Foxd1 than the lung, although the lung clearly contains Foxd1.
(TIF)

Figure S5 **Back gating strategy validating that the RFP/PECAM double positive cells in** ***Foxd1cre*** **CAG adult lung cells are not pericytes. A.** FACS plot showing the E15.5 *Foxd1cre*CAG RFP positive unstained cells (red dots) used to set the gates to determine the PECAM/RFP double positive cells. **B.** Representative FACS plot for unstained cells to set the gates for cells that are negative for CD73 and CD13. **C.** *Foxd1cre*CAG positive cells stained with PECAM showing the PECAM/RFP double positive cells (box). **C’.** Double positive cells from “C” co-stained with pericyte markers (CD73 and CD13) showing the vast majority of PECAM/RFP positive cells are pericyte marker negative.
(TIF)

Table S1 **Percentage of GFP labeled Foxd1-expressing renal stroma that co-expresses Flk1-positive endothelium at various developmental stages in** ***Foxd1EGFPcre*** **mouse kidney cells.**
(DOCX)

Table S2 **Percentage of GFP labeled Foxd1-expressing renal stroma that co-expresses PECAM-positive endothelium at various developmental stages in** ***Foxd1EGFPcre*** **mouse kidney cells.**
(DOCX)

Table S3 **Percentage of RFP permanently-labeled Foxd1 renal stromal derivatives that give rise to endothelium in** ***Foxd1cre*** **CAG** **adult kidneys.**
(DOCX)
