# Supplementary figures and images for: Endothelial Progenitors Exist within the Kidney and Lung Mesenchyme
Source: PLoS One. 2013 Jun 18;8(6):e65993. doi: 10.1371/journal.pone.0065993 (PMC3688860; doi:10.1371/journal.pone.0065993)

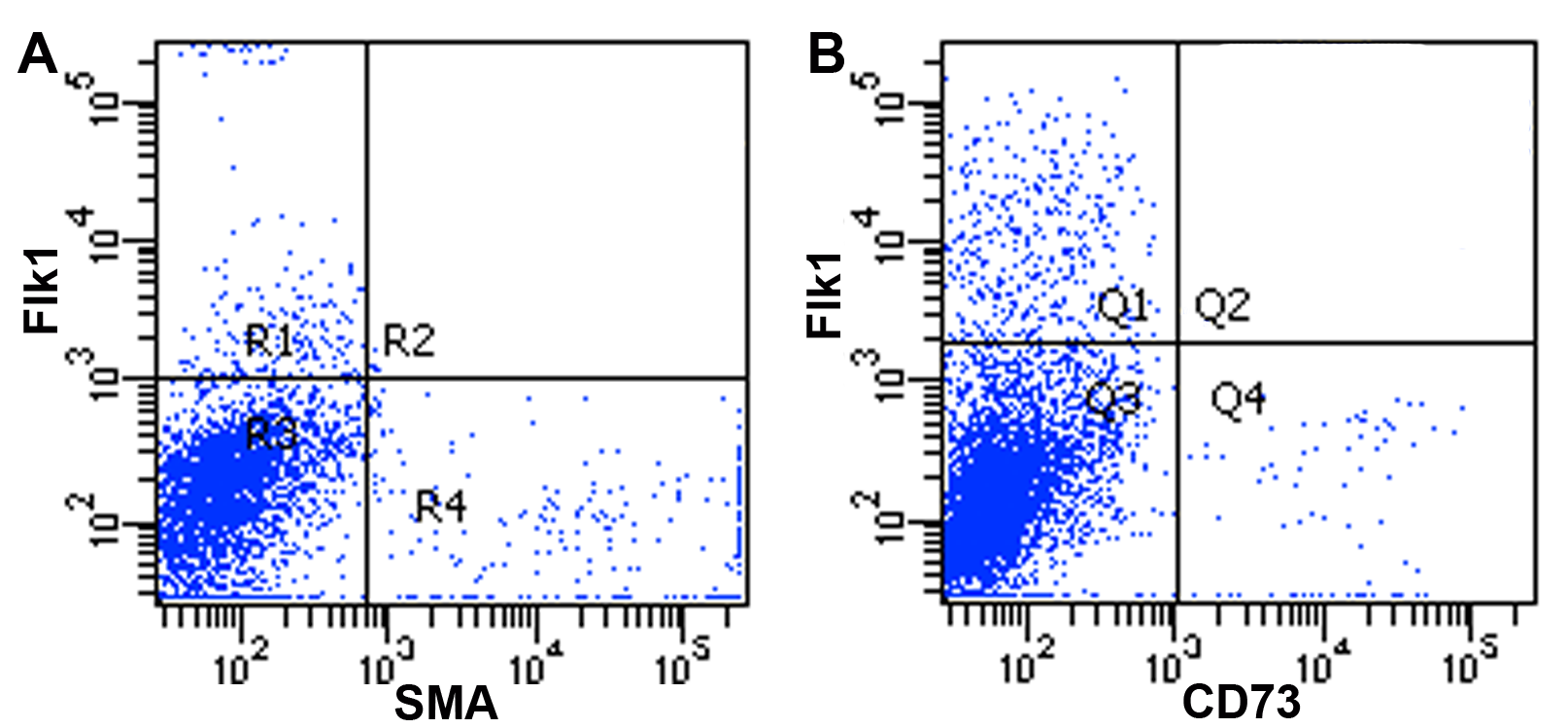

Supplement: Figure S1 — Non-overlapping expression of Flk1 and pericyte or muscle markers in embryonic kidney. A. Representative FACS plot from E15.5 kidney cells showing that there are no cells that co-express the endothelial marker Flk1 (Y axis) and the smooth muscle marker αSMA (Y axis) (panel R2). B. Representative FACS plot from E15.5 kidney cells showing that there are no cells that co-express the endothelial marker Flk1 (Y axis) and the pericyte marker CD73 (X axis) (panel Q2). (TIF) [file pone.0065993.s001.tif]

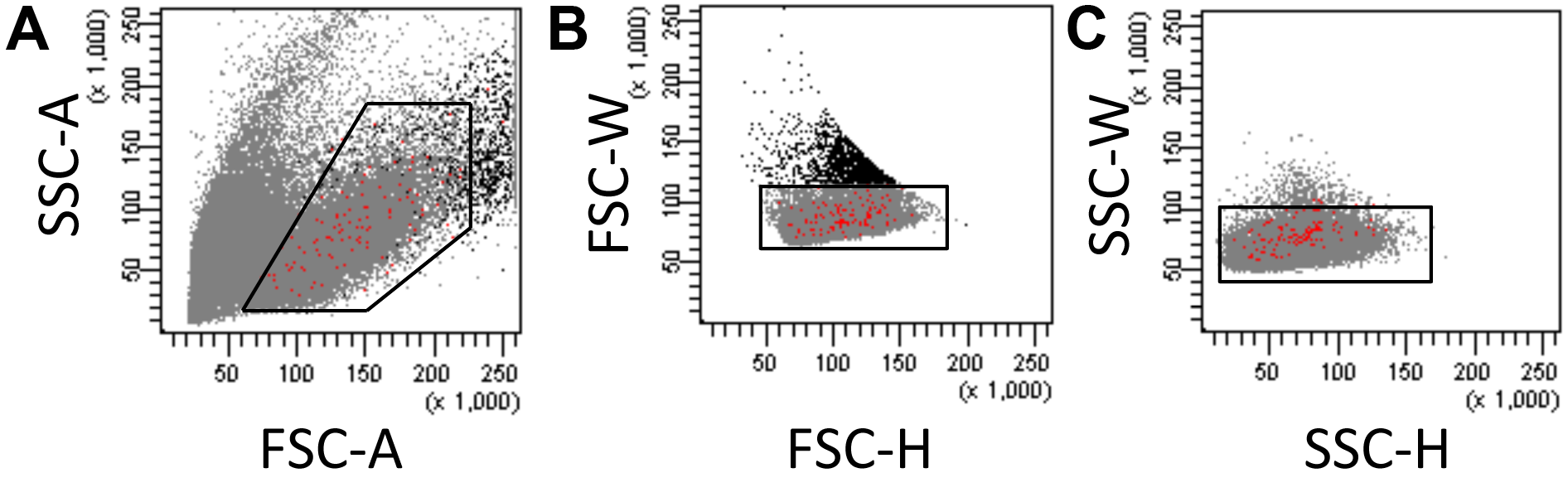

Supplement: Figure S2 — Gating out of debris and doublet discriminator. A. Representative FACS plot showing the gating strategy (box) used to eliminate the debris and red blood cells from E15.5 Foxd1creCAG kidney single cell suspensions. SSC-A = side scatter-area, FSC-A = forward scatter-area. B. Representative FACS plot showing the gating strategy used for forward scatter (box) to eliminate adherent cells. FSC-W = forward scatter width, FSC-H = forward scatter – height. C. Representative FACS plot showing the gating strategy used for side scatter (box) to eliminate adherent cells clinging together. SSC-W = side scatter – width, SSC-H = side scatter- height. The red dots in all panels represent the distribution of the double-positive GFP (Foxd1)/Flk1 cells within the population of gated cells. (TIF) [file pone.0065993.s002.tif]

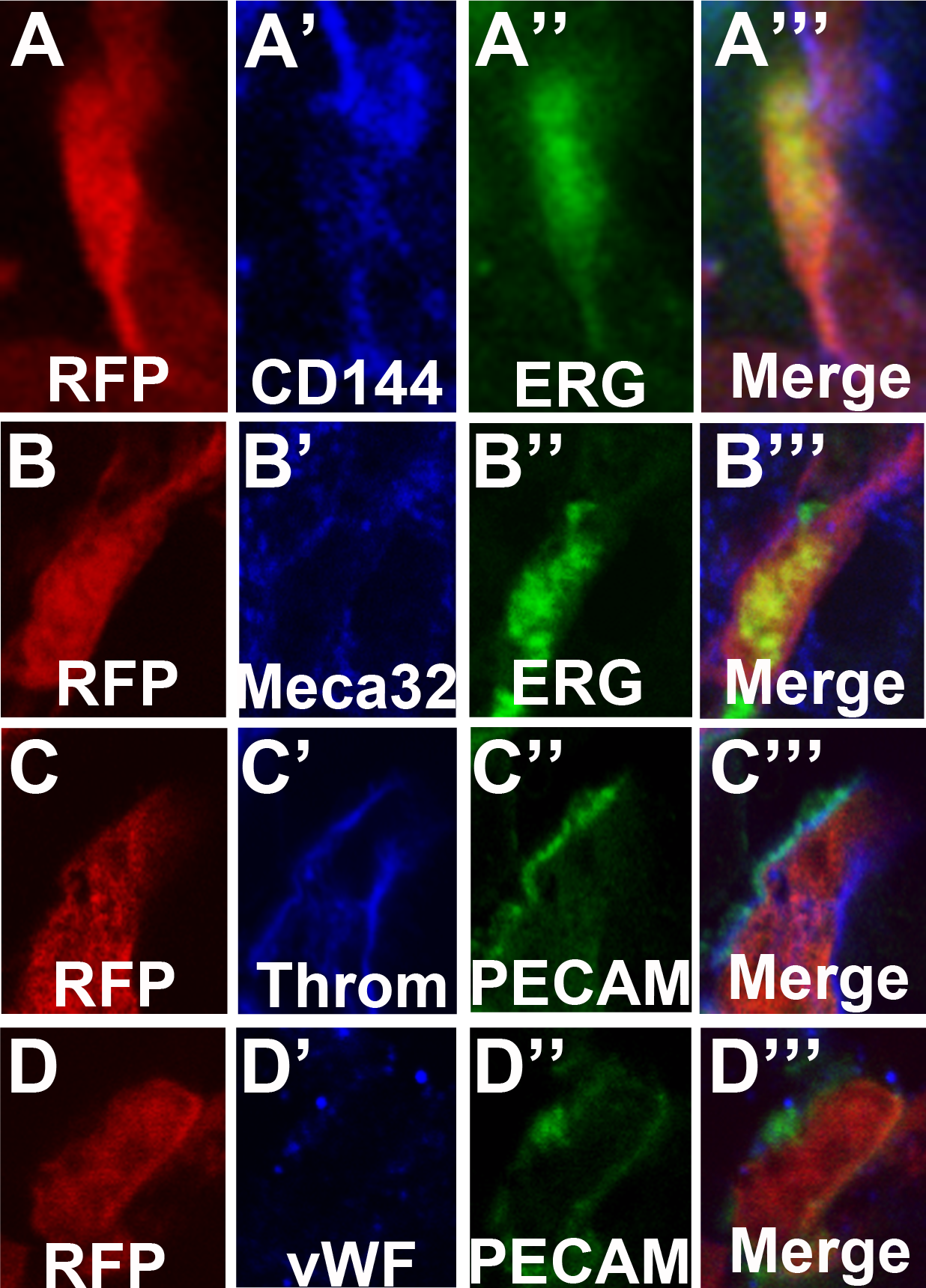

Supplement: Figure S3 — Immunofluorescence showing expression of multiple endothelial markers in a subset of Foxd1 derivatives in E18.5 peritubular capillaries. A–A’’’. RFP-positive cell derived from the Foxd1-expressing renal stroma (A) co-expresses the membrane endothelial marker CD144 (A’) and the nuclear endothelial marker Erg (A’’) as shown on the merged image (A’’’). B–B’’’. RFP-positive cell derived from the Foxd1-expressing renal stroma (B) co-expresses the membrane endothelial marker Meca32 (B’) and the nuclear endothelial marker Erg (B’’) as shown on the merged image (B’’’). C–C’’’. RFP-positive cell derived from the Foxd1-expressing renal stroma (C) co-expresses the membrane endothelial marker Thrombomodulin (C’) and the endothelial marker PECAM (C’’) as shown on the merged image (C’’’). D–D’’’. RFP-positive cell derived from the Foxd1-expressing renal stroma (D) co-expresses the Weibel-Palade bodies endothelial specific marker vWF (D’) and the endothelial marker PECAM (D’’) as shown on the merged image (D’’’). (TIF) [file pone.0065993.s003.tif]

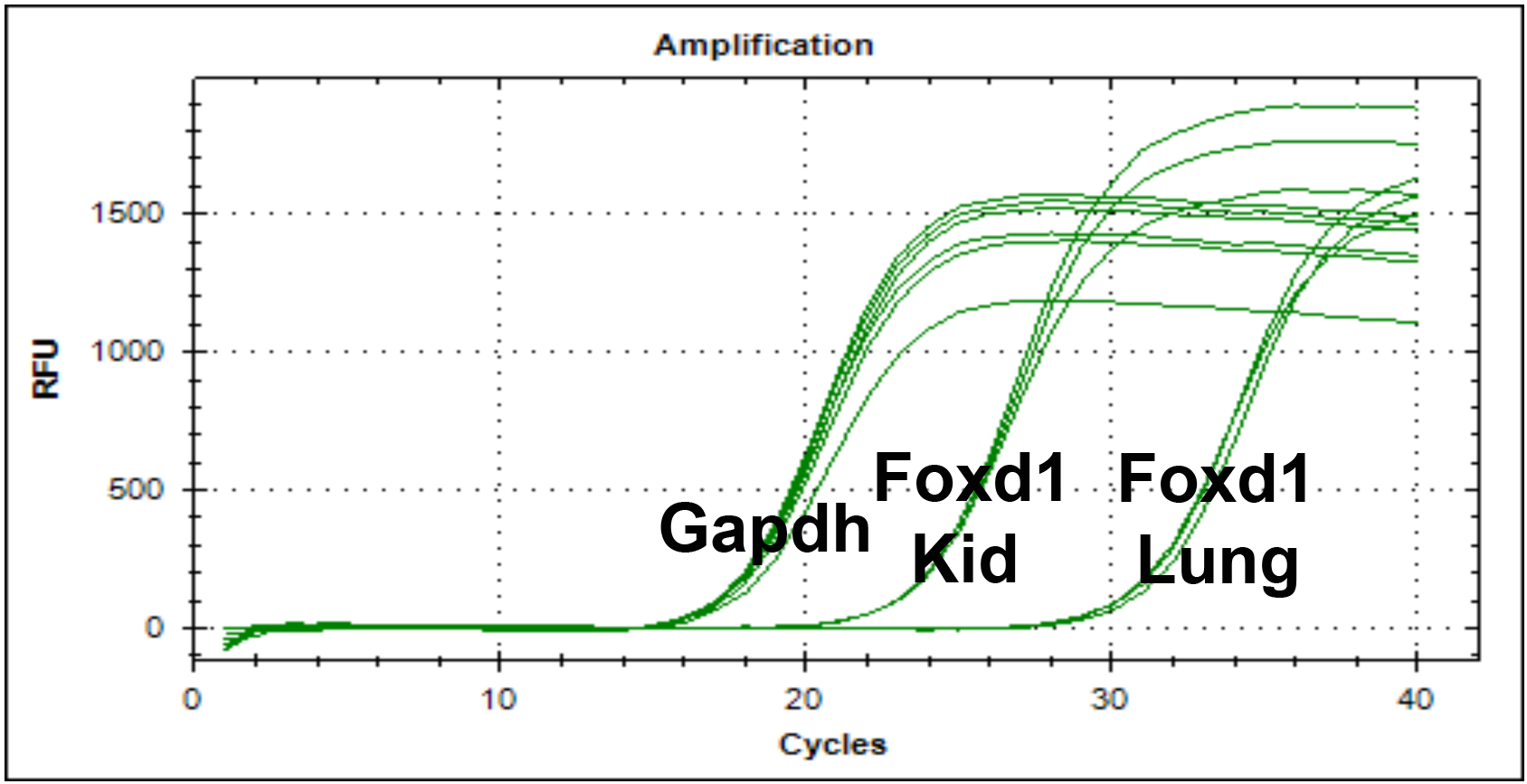

Supplement: Figure S4 — Representative real time PCR showing the presence of Foxd1 in the kidney and lung. Gapdh was used to generate the delta CT. Kidney samples showed expression after 23 cycles while the lung took 29 cycles showing that relatively the kidney contains more Foxd1 than the lung, although the lung clearly contains Foxd1. (TIF) [file pone.0065993.s004.tif]

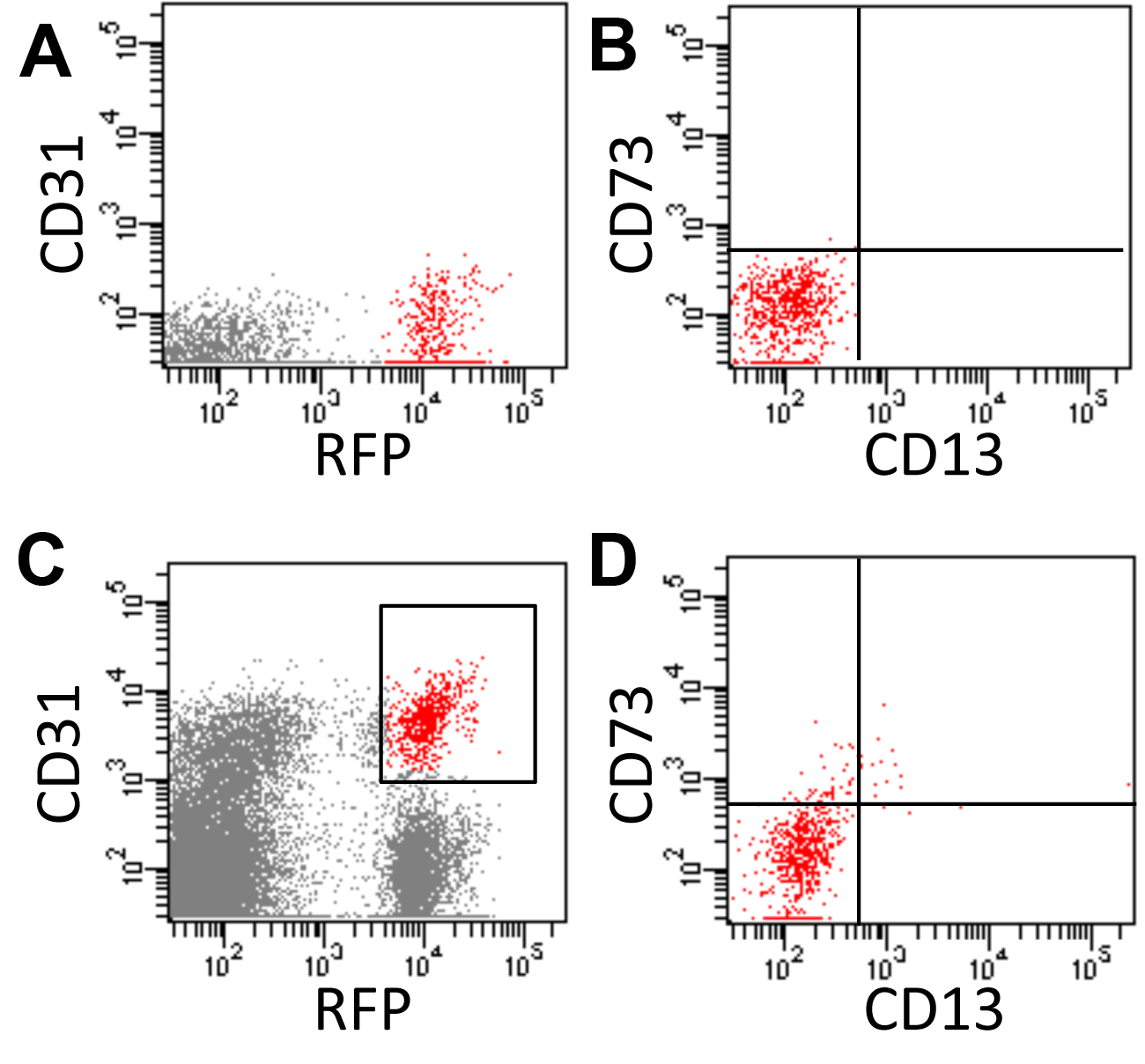

Supplement: Figure S5 — Back gating strategy validating that the RFP/PECAM double positive cells in Foxd1cre CAG adult lung cells are not pericytes. A. FACS plot showing the E15.5 Foxd1creCAG RFP positive unstained cells (red dots) used to set the gates to determine the PECAM/RFP double positive cells. B. Representative FACS plot for unstained cells to set the gates for cells that are negative for CD73 and CD13. C. Foxd1creCAG positive cells stained with PECAM showing the PECAM/RFP double positive cells (box). C’. Double positive cells from “C” co-stained with pericyte markers (CD73 and CD13) showing the vast majority of PECAM/RFP positive cells are pericyte marker negative. (TIF) [file pone.0065993.s005.tif]
