# Supplementary material for: Endothelial Progenitors Exist within the Kidney and Lung Mesenchyme
Source: PLoS One. 2013 Jun 18;8(6):e65993. doi: 10.1371/journal.pone.0065993 (PMC3688860; doi:10.1371/journal.pone.0065993)
Supplement: Table S1 — Percentage of GFP labeled Foxd1-expressing renal stroma that co-expresses Flk1-positive endothelium at various developmental stages in Foxd1EGFPcre mouse kidney cells. (DOCX) [file pone.0065993.s006.docx]

|  | GFP | | Flk1 | | GFP/Flk1 | |
| --- | --- | --- | --- | --- | --- | --- |
| E13.5 (n=3) | 4.1 ± 1.4 | | 6.9 ± 1.2 | | 0.3 ± 0.1 | |
| E15.5 (n=3) | 5.2 ± 2.1 | | 6.6 ± 1.7 | | 0.4 ± 0.2 | |
| E18.5 (n=3) | | 1.5 ± 0.4 | | 7.9 ± 2.0 | | 0.3 ± 0.1 |
| All values are means ± standard deviation  The % of stroma that co-expresses Flk1 is 6.8 (E13.5), 7.1 (E15.5) and 16.7 (E18.5)  The % of endothelium that expresses Foxd1 is 4.2 (E13.5), 5.7 (E15.5) and 3.7 (E18.5) | | | | | | |
