# Supplementary material for: Endothelial Progenitors Exist within the Kidney and Lung Mesenchyme
Source: PLoS One. 2013 Jun 18;8(6):e65993. doi: 10.1371/journal.pone.0065993 (PMC3688860; doi:10.1371/journal.pone.0065993)
Supplement: Table S2 — Percentage of GFP labeled Foxd1-expressing renal stroma that co-expresses PECAM-positive endothelium at various developmental stages in Foxd1EGFPcre mouse kidney cells. (DOCX) [file pone.0065993.s007.docx]

|  | GFP | | PECAM | | GFP/Flk1 | |
| --- | --- | --- | --- | --- | --- | --- |
| E13.5 (n=3) | 4.3 ± 0.3 | | 7.3 ± 0.6 | | 0.3 ± 0.1 | |
| E15.5 (n=3) | 3.4 ± 0.2 | | 6.6 ± 1.2 | | 0.4 ± 0.1 | |
| E18.5 (n=3) | | 1.8 ± 0.5 | | 8.6 ± 2.1 | | 0.3 ± 0.1 |
| All values are means ± standard deviation  The % of stroma that co-expresses PECAM is 6.5 (E13.5), 10.5 (E15.5) and 14.3 (E18.5)  The % of endothelium that expresses Foxd1 is 3.9 (E13.5), 5.7 (E15.5) and 3.4 (E18.5) | | | | | | |
