# Supplementary material for: Endothelial Progenitors Exist within the Kidney and Lung Mesenchyme
Source: PLoS One. 2013 Jun 18;8(6):e65993. doi: 10.1371/journal.pone.0065993 (PMC3688860; doi:10.1371/journal.pone.0065993)
Supplement: Table S3 — Percentage of RFP permanently-labeled Foxd1 renal stromal derivatives that give rise to endothelium in Foxd1cre CAG adult kidneys. (DOCX) [file pone.0065993.s008.docx]

|  | RFP | PECAM | RFP/PECAM |
| --- | --- | --- | --- |
| E18.5 (n=3) | 17.8 ± 3.4 | 5.1 ± 1.6 | 0.6 ± 0.2 |
| P30 (n=3) | 1.8 ± 0.2 | 2.8 ± 0.2 | 0.3 ± 0.1 |
| All values are means ± standard deviation  The % of stroma that gives rise to endothelium is 3.3 (E18.5) and 14.3 (P30)  The % of endothelium derived from the resident ECs is 10.5 (E15.5), and 9.7 (P30) | | | |
